# Metastasis in Neuroblastoma and Its Link to Autophagy

**DOI:** 10.3390/life13030818

**Published:** 2023-03-17

**Authors:** Leila Jahangiri

**Affiliations:** 1 School of Science and Technology, Nottingham Trent University, Clifton Site, Nottingham NG11 8NS, UK; leila.jahangiri@ntu.ac.uk; 2 Division of Cellular and Molecular Pathology, Department of Pathology, Addenbrooke’s Hospital, University of Cambridge, Cambridge CB2 0QQ, UK

**Keywords:** neuroblastoma, paediatric cancers, autophagy, metastasis

## Abstract

Neuroblastoma is a paediatric malignancy originating from the neural crest that commonly occurs in the abdomen and adrenal gland, leading to cancer-related deaths in children. Distant metastasis can be encountered at diagnosis in greater than half of these neuroblastoma patients. Autophagy, a self-degradative process, plays a key role in stress-related responses and the survival of cells and has been studied in neuroblastoma. Accordingly, in the early stages of metastasis, autophagy may suppress cancer cell invasion and migration, while its role may be reversed in later stages, and it may facilitate metastasis by enhancing cancer cell survival. To that end, a body of literature has revealed the mechanistic link between autophagy and metastasis in neuroblastoma in multiple steps of the metastatic cascade, including cancer cell invasion and migration, anoikis resistance, cancer cell dormancy, micrometastasis, and metastatic outbreak. This review aims to take a step forward and discuss the significance of multiple molecular players and compounds that may link autophagy to metastasis and map their function to various metastatic steps in neuroblastoma.

## 1. Introduction

Neuroblastoma (NB) accounts for close to 10% of all cancer cases in children but disproportionately leads to 15% of cancer-related deaths in this population of patients, equating to 1 in every 7000 live births [1]. The median age of diagnosis is usually 18 months, but it can be diagnosed in infants < 18 months, children 18 months–12 years, or adolescents older than 12 years of age [2,3]. NB is also regarded as one of the most common paediatric solid tumours relating to extracranial tissues and is thought to originate from the neural crest [3,4,5]. Furthermore, the site of presentation of this cancer varies from the abdomen, chest, neck, and pelvis, while over half of the patients will show metastasis to sites including the lymph nodes, bone, and bone marrow at the time of diagnosis [2,6,7,8].

Stages in NB can be categorised based on two systems: the international neuroblastoma risk group system (INRGSS) linked to image-defined risk factors (IDRF) and pre-treatment/surgery disease extent and comprise L1, L2, M and MS. On the other hand, the international neuroblastoma staging system (INSS) is linked to post-surgical disease extent and includes stages 1–4 and 4S (defined specifically in [3,9,10,11]). Briefly, the INRGSS L1 and L2 are both locoregional tumours, and L1 does not have IDRF, while L2 bears IDRF. Accordingly, IDRFs are defined as imaging surrogates of the tumour with regard to anatomical positions that predict a tumour resection success. M in this categorisation represents a widely spread disease, and MS (metastatic special) represents an age-specific (<18 months) localised disease (L1 and L2) linked to metastasis in locations including the skin, liver, and bone marrow (but not cortical bone) [3,9,10,11]. Further, the INSS stages 1 and 2 represent locoregional tumours having been completely or partially resected, respectively, and stage 3 denotes locoregional tumours of greater dimension which cross the midline and are deemed unresectable. Stages M and MS, however, represent distant metastasis and metastatic special, respectively. The latter denotes an age-specific (<12 months) category with a primary tumour of stage 1 and limited metastasis to the skin, liver and minimal bone marrow (but not cortical bone) [3,9,10,11].

Furthermore, patients can be stratified into low, intermediate, and high-risk, a classification based on various factors, including chromosomal alterations, MYCN status, stage, ploidy, age, and differentiation status, in addition to other criteria [3,12,13,14]. Concerning high-risk NB, MYCN amplification cases constitute approximately 40–50% of this group, and this genetic alteration is the strongest predictor of poor prognosis [3,12,13,14]. Additionally, high-risk NB may encompass two other subgroups, including alternating length of telomeres (ALT) (approximately 25%) and 23–31% of cases displaying telomere reverse transcriptase (*TERT*) gene rearrangements (e.g., rearrangement of 5p15.33, leading to strong enhancer insertion into *TERT* coding sequences) [9,15,16].

Risk groups also determine treatment stratification, ranging from surveillance to surgery for cases with low-risk stratification, response-adjusted doses of chemotherapy and surgery for intermediate-risk groups, and chemotherapy, surgery, radiotherapy, and myeloablative therapy for high-risk cases [13,14,17].

Tumour cell migration and invasion are key requirements for cancer metastasis and progression. Specifically, in the case of NB, metastasis to the lymph nodes, bone, and bone marrow accounts for most of the NB-related cancer progression and mortality [3,8,18]. To that effect, the clinical outcomes of NB patients with metastasis remain poor in contrast to patients with localised disease presentation that bear a more favourable overall survival (OS) projection [19]. OS is defined as the time post-diagnosis/treatment in which the patient is still alive. One good example of the comparison of prognoses is NB patients showing bone marrow metastasis compared to NB patients with no bone marrow metastasis, in which the former group displayed an OS of 35.87% compared to 87.7% in the latter cohort, respectively [20].

**Figure 1:**
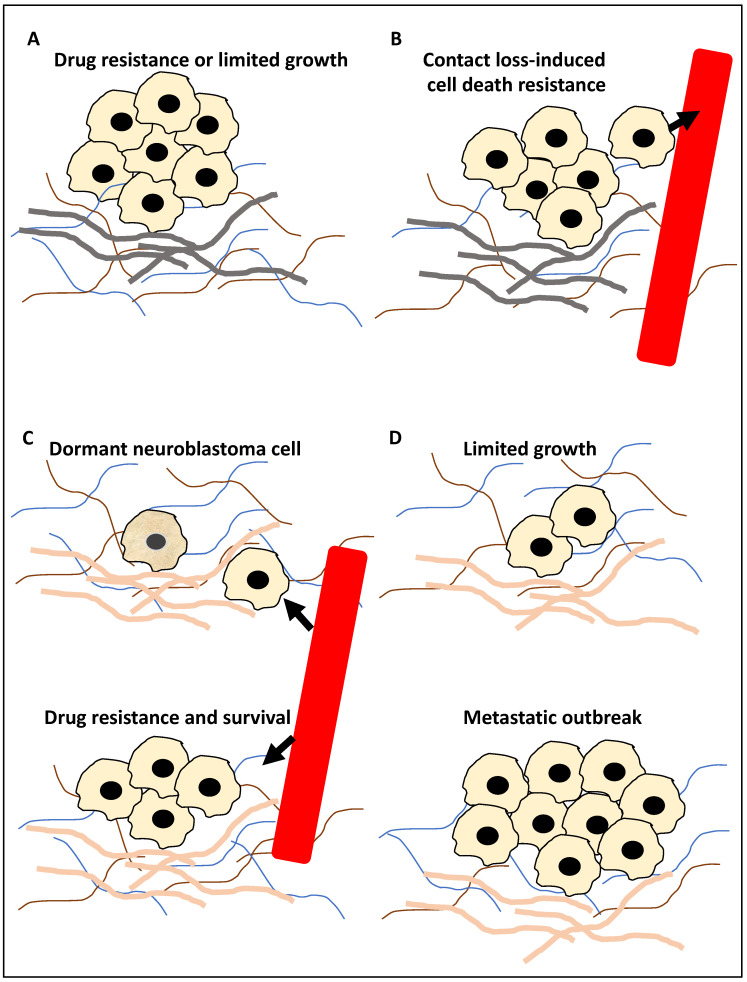
The roles of autophagy in metastasis. (**A**) The primary tumour cells (for example, neuroblastoma cells) during the in-situ step may be affected by autophagy; for example, autophagy may inhibit metastasis by limiting pro-metastasis inflammatory responses and limiting growth (−), or it can promote metastasis by increasing drug resistance (+). (**B**) Tumour cells (for example, neuroblastoma cells) can enter the circulation, constituting circulating tumour cells, and autophagy promotes resistance to anoikis (anoikis is triggered by a contact loss with the basement membrane), hence promoting metastasis (+). Arrowhead showing tumour cells entering the circulation. (**C**) At the extravasation and seeding step, the circulating tumour cells enter a distant site, and autophagy may trigger the onset of dormancy in the upper panel of (**C**) (the cell with a slightly darker yellow colour represents a dormant neuroblastoma cell) or survival in the new microenvironment (+) and drug resistance (+) in the bottom panel of (**C**). Arrowhead showing tumour cells exiting the circulation. (**D**) In the distant metastasis site, autophagy can limit the expansion of dormant tumour cells (+/−) in the upper panel of (**D**) or promote adaptation to this new environment, expansion and metastatic outbreak (+) in the bottom panel of (**D**). It is noteworthy that (+) and (−) signs in the text signify a positive or negative contribution of autophagy to metastasis, respectively, while the grey basement layer in (**A**,**B**) and pink basement layer in (**C**,**D**) represent distinct tumour microenvironments before and after migration, respectively.

From a molecular outlook, tumour cells must acclimate to harsh and stressful conditions to metastasise, enter the systemic circulation, disseminate, and eventually colonise a distant site [21]. During these processes, tumour cells will face cellular stressors, including the depletion of nutrition and oxygen resources and cell–cell or cell–basement membrane contact loss, and these factors may be inducers of autophagy, a tightly regulated self-preservation mechanism facilitated by lysosomal autolysis [22,23]. Given this background, it stands to reason that autophagy may be closely linked to the process of metastasis, and accordingly, autophagy plays a dual role in suppressing or promoting cancer progression due to the removal of damaged proteins and cellular components or conferring resistance to stress, respectively [23,24]. From a metastasis standpoint, the role of autophagy may be examined closer in 4 arbitrary steps comprising the in-situ primary tumour, intravasation, extravasation and seeding in new tissue, and finally, colonisation of distant sites [8,21,23,25,26] (Figure 1).

During step 1 (Figure 1A), autophagy may suppress metastasis by limiting tumour growth by reducing pro-metastasis immune cell infiltration. Equally, it can promote metastasis by promoting resistance to drug treatment, including TNF-related apoptosis-inducing ligand (TRAIL). In step 2 (Figure 1B), the tumour cells enter the circulation and thereby lose contact with the basement membrane, and the triggering of autophagy can suppress anoikis (cell death due to loss of cell–basement membrane contact). In step 3 (Figure 1C), surviving tumour cells (circulating tumour cells) may exit the circulation and seed within the distant tissue. Triggering autophagy may lead to resistance to drug treatment, including TRAIL, the survival of the tumour cells in the new microenvironment, or the onset of dormancy. Finally, in step 4 (Figure 1D), in the distant tissue, autophagy may limit the expansion of the dormant tumour cells or equally lead to a metastatic outbreak and expansion (reviewed in [8,21,23,25,26]). Given this background, this study aimed to take a step forward from describing mechanisms that link autophagy to metastasis and focus on examining the literature for molecular players and compounds that may link these processes, specifically in NB. In addition, for each molecular player and compound, the evidence supporting (a) its significance in NB, (b) the regulation of autophagy by this molecular player or compound in other cancers, and (c) whether it may be a suitable biomarker for predicting NB progression or may be used for NB treatment, will also be showcased and dissected.

## 2. Autophagy in NB Metastasis

### 2.1. The Molecular Basis of Autophagy

**Figure 2:**
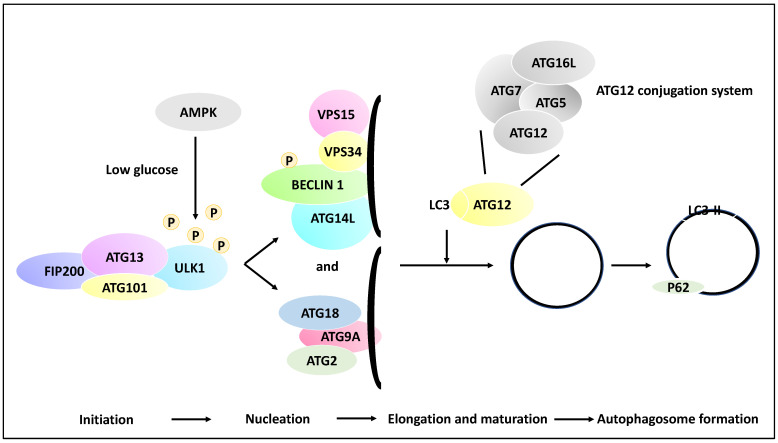
Multiple molecular steps of autophagy. A pre-autophagosomal structure is formed in the initiation stage and comprises ULK1, FIP200, ATG101 and ATG13, and in low glucose states, the ULK1 may receive activating phosphorylation via AMPK activity (whereas in the high-glucose state, ULK1 receives inhibitory phosphorylation due to mTOR and raptor activity, although this is not shown in the figure). ULK1 then activates the nucleation of the phagophore and the phosphorylation of Beclin 1. In the nucleation phase, ATG9A is recruited to the isolation membrane (phagophore), and the recruitment of ATG2 and ATG18 ensues. In addition, the isolation membrane is coated with VPS34, VPS15, ATG14L, and Beclin 1. Further, in the elongation and maturation phase, the processing of LC3 and ATG12 conjugation systems leads to the elongation of the phagophore and the formation of autophagosomes. It is noteworthy that the ATG12 conjugation system comprises ATG12, ATG7, ATG16L and ATG5 proteins. These processes contribute to the coating of the autophagosome with proteins such as LC3-II and P62, while the formed autophagosome may fuse with a lysosome in later stages.

Autophagic processes involve a myriad of over 30 genes termed autophagy-related genes (ATGs) that contribute to the main steps of autophagy, including the initiation, the process of the phagophore nucleation, the elongation/maturation of a phagophore and autophagosome formation, and finally, the fusion of the lysosomes with the autophagosome [27,28]. To that end, phagophore initiation is orchestrated by the release of ULK1 from the ULK1 complex (ULK1/ATG13/FIP200/ATG101), and this process is dependent on the mTOR and AMPK signalling pathways (the induction step). For example, in low-glucose states, the ULK1 function may be altered by receiving an activating phosphorylation modification from AMP-activated kinase (AMPK). In contrast, in a high-glucose state, ULK1 receives inhibitory phosphorylation due to the activity of mTOR and raptor [29]. ULK1, post-activating phosphorylation, will activate the nucleation of the phagophore by phosphorylating Beclin 1. The recruitment of ATG9A and its associated proteins to the phagophore complex (nucleation) also ensues. These events lead to the formation of a phagophore (isolation membrane) coated by a complex of proteins inclusive of VPS15, VPS34, ATG14L and Beclin 1 (also known as the PI3K complex). Autophagosome elongation and maturation are mediated through the activation of ATG12 and cleavage of microtubule-associated protein light chain 3 (LC3) conjugation systems. As a result of these processes, the autophagosome is then coated with LC3-II-bound P62, and since P62 is degraded by the autolysosomes, hence the reduction of P62 is an indicator of autophagy progression. Finally, LC3-II levels are indicative of autophagosome formation since LC3-I is converted to LC3-II through lipidation processes [21,30,31] (Figure 2).

### 2.2. Molecular Players and Compounds Linking Autophagy to Metastasis

The following sections will investigate 11 molecular players or compounds linking autophagy to metastasis in NB in several categories, including autophagy players, non-coding RNA and compounds. This information has been summarised in Table 1.

#### 2.2.1. Mediators of Autophagy and Their Link to NB Metastasis

UNC-51-like kinase1 (ULK1) is a serine/threonine kinase and forms a complex with ATG13, ATG101 and FIP200. AMPK can mark ULK1 with activating phosphorylation under energy and nutrient depletion conditions, and this will activate the nucleation of the phagophore by phosphorylating Beclin 1 and thereby advance the process of autophagy [43]. Therefore, ULK1 is a kinase that regulates the early steps of autophagy. Given this background, Dower and colleagues exposed a panel of NB cell lines, including SH-SY5Y and SK-N-AS cell lines, to a small molecule inhibitor of ULK1, SBI-0206965 [32]. This compound reduced autophagic flux in SK-N-AS cells under starvation conditions, decreased ULK1 and increased P62 protein levels (a protein accumulated when autophagy is inhibited) [44]. Autophagic flux is defined as the molecular process of autophagy, including autophagosome formation followed by the lysis of macromolecules [23]. In addition, this treatment led to a decreased LC3-II accumulation in the presence of bafilomycin A1 (a molecule that inhibits autophagosome-lysosome fusion) [32]. These proof-of-principle experiments revealed that SBI-0206965 reduced autophagic flux and LC3 lipidation. In addition, under both normal and starvation conditions, SBI-0206965 treatment led to the upregulation of cleaved poly-ADP ribose polymerase (PARP) and caspase-3 and consequently apoptosis (and annexin-V staining), while cell viability was decreased [32]. The effect on cytotoxicity was more profound under starvation conditions, presumably due to the dependence of NB cells on autophagy for survival under these conditions, hence collectively suggesting that the small molecule inhibitor suppressed autophagy but promoted apoptosis [32].

Further, the authors used a genetic model, SK-N-AS cells expressing a dominant-negative ULK1 gene (i.e., *dnULK1^K46N^* allele forming *dnULK1*-SK-N-AS cells), to show similar effects to SBI-0206965 on apoptosis. In evidence, dnULK1 increased P62, cleaved caspase-3 and PARP. In addition, the activity of caspase-3/7/8 increased, as did the percentage of annexin-V-expressing cells [32].

Consistently, the xenografting of SK-N-AS cells expressing stable *dnULK1* or empty vectors to the NOD/SCID-gamma (NSG) mouse model was coupled with monitoring tumour growth using a luciferase reporter. This approach showed that *dnULK1*-SK-N-AS xenograft grew much slower, displayed P62 accumulation, and increased PARP and caspase-3 levels compared to empty vector controls [32]. Further, using a metastatic burden mouse model, SK-N-AS cells expressing stable *dnULK1* or empty vector were xenografted to the bloodstream of mice via their tail vein; the former group survived for a significantly longer period and also displayed reduced liver metastasis burden, while the empty vector group displayed enlarged and fluid-filled livers [32].

**Figure 3:**
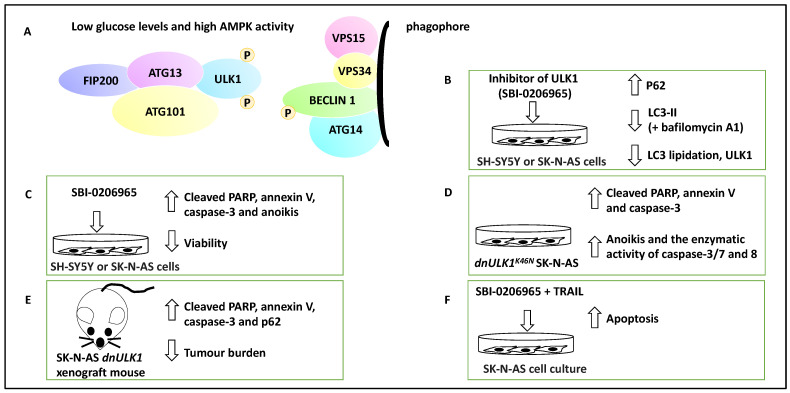
The contribution of ULK1 to NB apoptosis and metastasis. (**A**) ULK1 forms complexes with ATG13, ATG101 and FIP200. ULK1 can be activated by AMPK via activating phosphorylation when glucose levels are low. ULK1 then can contribute to the activation of nucleation of the phagophore and the phosphorylation of Beclin 1. (**B**) The addition of SBI-0206965, a small molecule inhibitor of ULK1, led to the inhibition of autophagy marked by an increase in P62, a decrease in ULK1, LC3 lipidation, and LC3-II levels in the presence of bafilomycin A1. Therefore, SBI-0206965 decreased autophagic flux. (**C**) SBI-0206965 treatment in NB cell lines led to the upregulation of PARP and caspase-3, while cell viability was decreased, suggesting that the inhibition of ULK1 could lead to anoikis (hence ULK1 leads to anoikis resistance and perhaps metastasis). (**D**) The genetic inhibition model, SK-N-AS cells expressing a dominant-negative ULK1 gene (e.g., *dnULK1^K46N^*), similar to SBI-0206965, led to increased cleaved PARP, annexin-V, caspase-3 and enhanced enzymatic activity of caspase-3/7 and 8 and may also promote anoikis (hence the presence of ULK1 can lead to anoikis resistance). (**E**) Xenografting of SK-N-AS cells expressing stable *dnULK1* led to P62 accumulation, increased PARP and caspase-3 levels, and a reduced metastasis burden in the liver in this group compared to their control counterparts. (**F**) TRAIL and SBI-0206965 combination in SK-N-AS cells led to increased apoptosis. TRAIL treatment, per se, increased autophagic flux, suggesting that perhaps autophagy has been upregulated to counter TRAIL-mediated apoptosis levels.

Regarding anoikis, which is defined as a form of apoptosis due to prolonged detachment of cells from their basement membrane and extracellular matrix, both dnULK1 and SBI-0206965 treatments in SK-N-AS cells lead to increased caspase-3/7/8 activation, annexin-V and cleaved PARP, suggesting that the inhibition and targeting of ULK1 could perhaps lead to increased anoikis and result in metastasis suppression [32]. In addition, potential synergism between TRAIL and SBI-0206965 was investigated in SK-N-AS cells, and this combination led to increased apoptosis and TRAIL-sensitised SK-N-AS cells to SBI-0206965. TRAIL treatment, per se, increased autophagic flux, suggesting that perhaps autophagy has been upregulated to alleviate TRAIL-mediated apoptosis levels [32]. This study highlighted the therapeutic potential of ULK1 in NB and how autophagic flux may be linked to the metastasis of tumour cells (Figure 3).

#### 2.2.2. Non-Coding RNA with Oncogenic Roles Linked to Autophagy in NB Metastasis

Many studies have investigated the role of long non-coding RNAs (lncRNAs) in the NB pathogenesis and progression [45,46,47]. As evidence, in a study, the role of NORAD, a lncRNA, in the pathogenesis of NB was investigated, and the effect of this lncRNA on the proliferation, apoptosis, autophagy and metastasis was assessed [33]. Accordingly, NORAD was upregulated in NB tissues compared to matched healthy tissues, while the expression of NORAD was associated with advanced INSS stage and metastasis. In addition, the knockdown of NORAD using siRNA led to proliferation reduction in NB cell lines, including SK-N-SH, and IMR-32 [33]. In addition, NORAD knockdown led to the promotion of apoptosis and both aspects were validated on a protein level whereby NORAD knockdown reduced PCNA, cyclin D1 and Bcl-2 protein, while Bax protein was upregulated (PCNA and cyclin D testing proliferation, Bax and Bcl-2 testing apoptosis, and Bcl-2 is an apoptosis inhibitor). Invasion and transwell migration assays were also utilised to show that the knockdown of NORAD led to reduced migration and invasion capacity of the NB cells [33]. Furthermore, pertinent to the link between NORAD and autophagy, the authors observed that the knockdown of NORAD also increased the protein levels of Beclin 1, LC3-II/I ratio and ATG5 and a reduction of P62, suggesting that autophagic cell death was triggered as a result of NORAD depletion. Having established the baseline effect of knocking down NORAD, the authors attempted to establish the effect of NORAD knockdown on the chemoresistance of NB cell lines, inclusive of SK-N-SH and IMR-32 treated with Doxorubicin (DOX) [33]. This revealed a statistically significant reduction in NB proliferation, metastasis, and DOX resistance (evident by a decrease in the half-maximal inhibitory concentration (IC50) for NORAD-depleted samples). NORAD, therefore, effectively promoted resistance to DOX, metastasis and proliferation, whereas it inhibited autophagy and apoptosis [33].

Furthermore, using Starbase software, the authors revealed that *miR-144-3p* was a target of NORAD, and this was further verified using reporter assays, while it was also revealed that the overexpression of NORAD led to *miR-144-3p* downregulation [33]. This link prompted the authors to determine the mechanistic basis of NORAD-based cancer progression and DOX resistance through the potential sponging effect of *miR-144-3p* activity. Initially, the authors revealed that the depletion of NORAD led to enhanced *miR-144-3p* levels [33]. Further, the depletion of *miR-144-3p* (using anti-*miR-144-3p*) reduced the suppressive effects of siRNA-mediated NORAD depletion on NB cell proliferation, migration, metastasis, and DOX resistance; hence the NORAD exerted its oncogenic effects through the negative regulation of *miR-144-3p.* Furthermore, histone deacetylase 8 (HDAC8) was shown to be a target of *miR-144-3p* in NB since the protein levels of HDAC8 were increased or decreased as a result of anti-*miR-144-3p* treatment or *miR-144-3p* accumulation, respectively. Furthermore, NORAD depletion led to reduced HDAC8 expression while anti-*miR-144-3p* treatment recovered the expression of HDAC8; hence HDAC8 was regulated through the NORAD/*miR-144-3p* axis [33].

**Figure 4:**
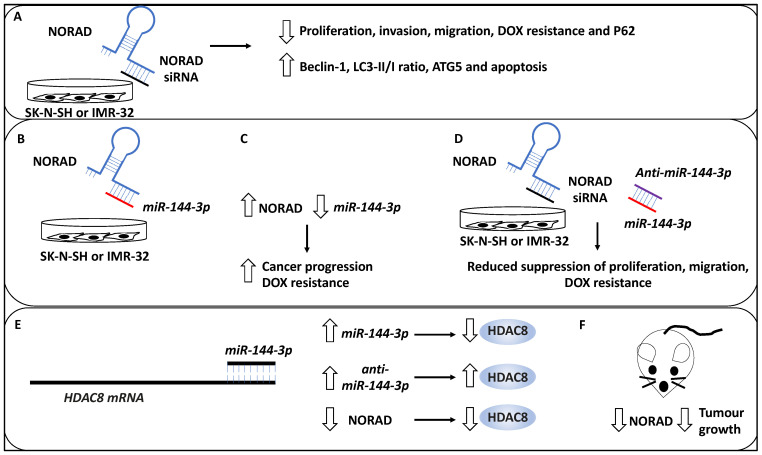
NORAD-mediated NB progression. (**A**) The knockdown of lncRNA NORAD using siRNAs reduces proliferation, invasion, and DOX resistance in NB cell lines, including SK-N-SH and IMR-32. In addition, NORAD knockdown may lead to P62 downregulation and increased levels of Beclin 1, ATG5, LC3-II/LC3-I (LC3-II/1), and enhanced apoptosis. (**B**) *miR-144-3p* was a target of NORAD, depicted by the binding of NORAD (blue) to *miR-144-3p* (red). (**C**) Further, the overexpression of NORAD led to the downregulation of *miR-144-3p*, resulting in cancer progression and DOX resistance. (**D**) The depletion of *miR-144-3p* (red) using *anti-miR-144-3p* (purple) reduced the suppressive effects of siRNA-mediated NORAD depletion on NB cell proliferation, migration, metastasis, and DOX resistance (NORAD and NORAD siRNA have been depicted in blue and black colours, respectively). (**E**) Histone deacetylase 8 (HDAC8) was shown to be a target of *miR-144-3p* in NB. *miR-144-3p* expression (accumulation) led to the downregulation of protein levels of HDAC8, while *anti-miR-144-3p* treatment led to HDAC8 upregulation. In addition, NORAD depletion led to reduced HDAC8 expression, collectively suggesting HDAC8 was regulated through the NORAD/*miR-144-3p* axis. (**F**) NORAD depletion in a murine xenograft model led to reduced tumour growth.

Finally, the authors also tested the effect of NORAD depletion on a murine xenograft model, showing that this treatment led to reduced tumour growth. Measuring protein levels in these in vivo samples also showed the reduction in NORAD and HDAC8 and the increase in *miR-144-3p* levels in NORAD-depleted samples. Overall, NORAD promoted proliferation, metastasis, and drug resistance, while it inhibited autophagy and apoptosis [33] (Figure 4).

Other oncogenes have also been studied in NB, including the role of hsa_circ_0013401, a circular RNA (circRNA), in proliferation, apoptosis, autophagy, and migration in this cancer. CircRNAs lack a cap structure at the 5′ ends and can therefore form circular structures. As evidence, hsa_circ_0013401 knockdown in NB cell lines, including SH-SY5Y and SK-N-BE, led to reduced proliferation and migration while apoptosis and autophagy were promoted [34]. The effect of hsa_circ_0013401 knockdown on apoptosis and autophagy was evident since the authors revealed an increase in the protein levels of Bax, cleaved caspase-3, LC3B-II/I ratio and Beclin 1. Accordingly, *miR-195* was a target of hsa_circ_0013401, while PAK2 was a target of *miR-195* (tested through reporter assays) and in a mouse xenograft model, hsa_circ_0013401 enhanced tumour formation and progression through the regulatory axis of *miR-195*/PAK2 [34]. In addition, the role of another lncRNA, SNHG16, in NB metastasis was investigated. This lncRNA was linked to poor clinical outcomes in NB patients. Furthermore, SNHG16 knockdown led to reduced proliferation and migration in NB cell lines (for example, SK-N-SH and IMR-32) and animal models [35]. This lncRNA formed a regulatory loop with ATG5 and *miR-542-3p* through which its effects on proliferation, migration and autophagy were implemented. Mechanistically, SNHG16 upregulated ATG5 and induced a sponging effect on *miR-542-3p* [35].

#### 2.2.3. Non-Coding RNA with Tumour Suppressor Roles That Affect Autophagy in NB Metastasis

In another study, the role of MEG3 a nucleus-based lncRNA, in NB was investigated since this lncRNA was negatively linked with the NB INSS stage [36]. In addition, MEG3 was positively linked to NB patient survival and negatively linked to the unfavourable clinical characteristics of these patients. For example, NB patients with higher MEG3 expression displayed a greater 5-year event-free survival (EFS) along with overall survival, while the lower expression of MEG3 was linked to NB disease progression [36]. EFS refers to the period after treatment in which the patient has not suffered from any events which were intended to be prevented by the treatment. Further, the effect of the upregulation of MEG3 in SK-N-BE2, SK-N-AS and SH-SY5Y cell lines was assessed by assays including colony formation, EDU and CCK-8 and these experiments showed that MEG3 suppressed NB cell proliferation and reduced colony formation capacity [36]. Using transwell and wound-healing assays, MEG3 was shown to suppress cell migration and invasion [36]. Gene ontology analyses revealed that MEG3 was involved in metabolic and catalytic activities in addition to autophagy and mTOR signalling pathways. The link between MEG3 and autophagy was also tested by upregulating MEG3, resulting in reduced Beclin 1, ATG3, ATG12 and LC3-II/LC3-I ratios. In addition, the link between autophagy and mTOR was expected since mTOR is a known autophagy regulator; however, what was not fully understood was whether MEG3 regulated autophagy in an mTOR-linked fashion in NB, and to address this, two pieces of evidence were generated [36]. Firstly, the overexpression of MEG3 inactivated mTORC1, implying that MEG3 may have affected autophagy in an mTOR-independent fashion. This statement was fortified by observing that rapamycin (an mTOR pathway inhibitor) did not affect NB autophagy (but affected migration). Secondly, the FOXO1 inhibitor, AS1842856, suppressed autophagy (e.g., lowered LC3), while the overexpression of MEG3 suppressed FOXO1 expression. This evidence collectively led to the conclusion that MEG3 regulated autophagy through FOXO1 modulation but not through the mTOR signalling pathway [36].

**Figure 5:**
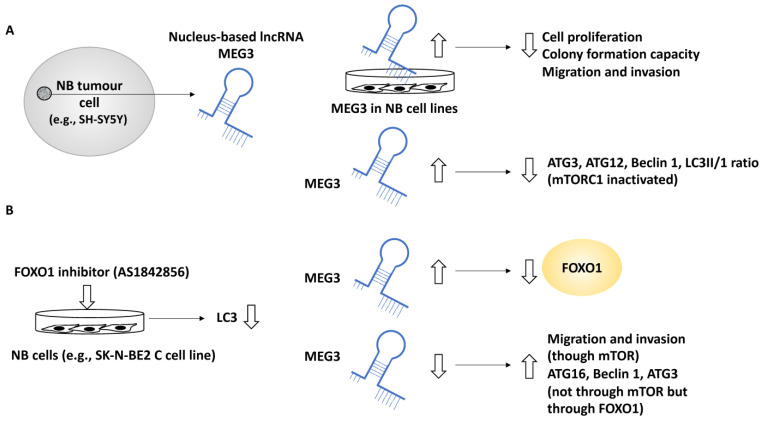
The role of MEG3 in regulating autophagy in NB metastasis. (**A**) MEG3, a nucleus-based lncRNA, was tested in NB cell lines, including SK-N-BE2, SK-N-AS, and SH-SY5Y. The overexpression of MEG3 led to reduced cell proliferation, colony formation, migration, and invasion capacity. In addition, MEG3 overexpression reduced autophagy protein levels, including ATG3, ATG12, and Beclin 1, but this effect was not implemented through mTORC1 since in MEG3-overexpressing NB cells, mTORC1 was inactivated. (**B**) A FOXO1 inhibitor (AS1842856) mimicked MEG3 overexpression and led to reduced LC3 protein levels (suppression of autophagy), while the overexpression of MEG3 suppressed FOXO1, suggesting perhaps that MEG3 attenuated autophagy through FOXO1 regulation. MEG3 downregulation led to enhanced levels of ATG16, ATG3, and Beclin 1 (in an mTOR-independent manner), while low levels of MEG3 enhanced migration through mTOR signalling.

Finally, the downregulation of MEG3 (by using a MEG3 silencer) led to NB malignant characteristics, including enhanced proliferation, migration, and invasion established using transwell assays in an mTOR-dependent fashion, while the silencing of MEG3 increased autophagy markers, including ATG16, Beclin 1 and ATG3, collectively bringing into light the role of MEG3 as a negative regulator of autophagy and metastasis in NB [36] (Figure 5).

Other tumour suppressor non-coding RNAs, including *miR-34a*, have also been investigated in the context of NB metastasis [37]. This non-coding RNA was suppressed in NB cell lines (for example, SH-SY5Y and SK-N-SH) and patient tissue linked with reduced viability and survival, respectively. *miR-34a* targeted ATG5 and thereby suppressed proliferation, apoptosis, metastasis and autophagy. This was evident since restoring ATG5 rescued the inhibitory effect of *miR-34a* on these processes [37].

#### 2.2.4. Compounds and Small Molecule Inhibitors That Link Autophagy in NB Metastasis

**Figure 6:**
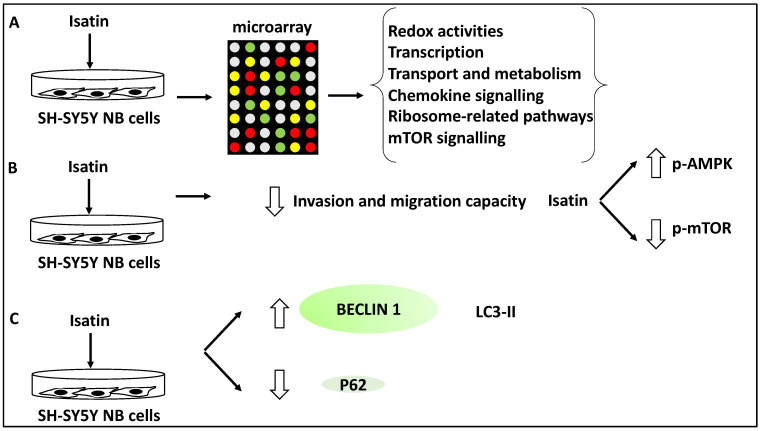
Isatin reduced the invasion and metastasis capacity of NB cells. (**A**) SH-SY5Y cells were treated with Isatin and were subjected to microarray analysis, revealing the differential expression of genes involved in redox activities, transcription, transport, metabolism, chemokine and mTOR signalling, and ribosome-related pathways. (**B**) Isatin treatment led to the reduced invasion and migration capacity of SH-SY5Y NB cells through the inhibition of phosphorylated-mTOR (p-mTOR) and the increase of phosphorylated-AMPK (p-AMPK) (since AMPK inhibits mTOR). (**C**) Isatin treatment led to the upregulation of LC3-II and Beclin1 and the downregulation of P62.

In another study, the effect of Isatin (a derivative of indirubin with anti-tumour properties) on SH-SY5Y NB cells was investigated using microarray analyses revealing 429 differentially expressed genes. Further, gene ontology analysis revealed that these differentially expressed genes displayed roles in redox activities, transcription, transport and metabolism [38]. Further, KEGG analysis of the differentially expressed gene list revealed the involvement of Isatin-regulated genes in chemokine and mTOR signalling and ribosome-related pathways [38]. A subset of genes relating to mTOR signalling, including DDIT4, RHEB, EIF4EBP1 and RPS6KB1, were taken forward for further analysis, and the differential expression of these genes was verified using RT-qPCR [38]. The link between the mTOR pathways and NB metastasis and invasion was also investigated in SH-SY5Y cells. Isatin reduced the invasion capacity of SH-SY5Y, tested through migration and invasion assays [38]. From a mechanistic viewpoint, Isatin reduced metastasis through the inhibition of mTOR phosphorylation while it increased the phosphorylation levels of AMPK, the inhibitor kinase of mTOR. Since autophagy is linked to mTOR signalling, the authors also tested the expression levels of LC3-II, Beclin1, and P62. Isatin treatment led to LC3-II and Beclin1 upregulation, and P62 downregulation, suggesting the induction of autophagy [38]. In effect, Isatin suppressed NB metastasis while triggering higher levels of autophagy markers and mTOR dysregulation. This suggested that autophagy is closely linked to metastasis in NB. Overall, it could be argued that Isatin exerted inhibitory effects on NB metastasis through autophagy-related and mTOR signalling pathways [38] (Figure 6).

The role of apatinib, a vascular endothelial growth factor receptor-2 (VEGFR-2) small molecule inhibitor in inducing cell cycle arrest and apoptosis in a panel of NB cells, including BE(2)-M17, IMR-32 and SH-SY5Y, was assessed [39]. As evidence, this study used a CCK-8 and colony-forming assays to test for viability and proliferation across different doses of apatinib, and this compound was shown to reduce NB cell viability, proliferation, and colony formation. In addition, apatinib reduced the expression of Ki-67, a proliferation marker, in these cells. Further, apatinib treatment led to the arrest of these cells in the G0/G1 phase and the increase in apoptotic cells in both BE(2)-M17 and SH-SY5Y cell lines; this was evident since the levels of both Bcl-2/Bax ratio (an apoptosis direction indicator), and cyclin D1 levels were reduced [39]. Thus, apatinib induced apoptosis and cell cycle arrest while inhibiting cell viability and colony formation. Furthermore, using both transwell and wound healing assays in BE(2)-M17 and SH-SY5Y cell lines, apatinib significantly reduced migration potential in these cells. Mechanistically, bioinformatic analyses showed that apatinib could affect the PI3K/AKT and MAPK/ERK pathways, while this was also established in vitro since this molecule downregulated p-AKT, p-mTOR and p-ERK protein levels [39]. Finally, the LC3B-II/I ratio (LC3B-II is an indicator of the number of autophagosomes, while LC3B is the main isoform of LC3) and ATG5 protein levels were increased as a result of apatinib treatment of SH-SY5Y and BE(2)-M17 NB cells [39,48].

**Figure 7:**
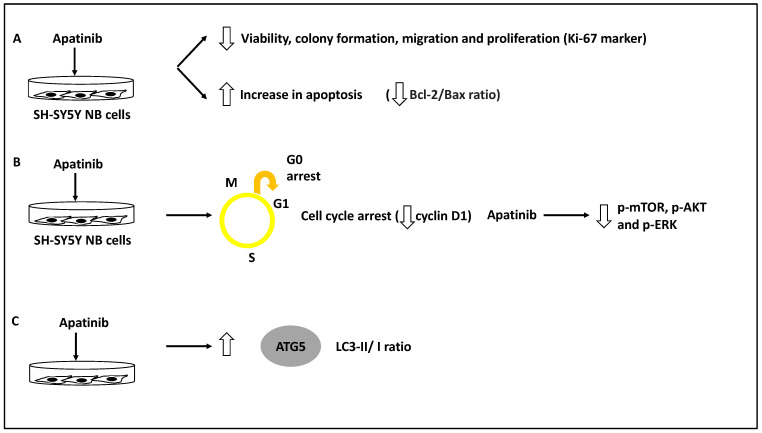
Apatinib reduced the invasion and metastasis capacity of NB cells. (**A**) NB cell lines inclusive of BE(2)-M17, IMR-32 and SH-SY5Y were treated with apatinib leading to reduced viability, colony formation, migration and proliferation of these cells while increasing apoptosis rate (reduced Bcl-2/Bax ratio). (**B**) Apatinib treatment led to cell cycle arrest (reduced cyclin D1 levels) while decreasing phosphorylated mTOR, AKT, and ERK (p-mTOR, p-AKT and p-ERK, respectively). (**C**) LC3-II/I and ATG5 protein levels were increased as a result of apatinib treatment, revealing autophagy activation.

It could be argued this study revealed that apatinib treatment could induce apoptosis and autophagy while suppressing metastasis through the PI3K/AKT/mTOR and MAPK/ERK signalling axis [39]. However, the direct link between the suppression of VEGFR2 by apatinib and the in vivo reduction of metastasis was not investigated, but since apatinib inhibits p-mTOR, this may be the logical explanation for this compound’s capacity to reduce NB migration [39] (Figure 7). In agreement with these studies, Honokiol was also shown to induce apoptosis and autophagy (for example, it increased LC3-II levels in neuro-2a NB cell line) and reduce migration, and these effects were linked to the PI3K/Akt/mTOR pathway [40].

## 3. Discussion; Molecular Players and Compounds That May Link NB Metastasis to Autophagy

### 3.1. Mapping Various Molecular Players and Compounds to Metastasis Steps

This study catalogued and summarised the evidence underpinning the molecular players linking autophagy and metastasis in NB. Tumour cell migration and metastasis are largely viewed as essential steps in metastasis. Consistently, most NB-related deaths are linked to tumour metastasis of the bone, bone marrow, and lymph nodes, and over 50% of patients will display metastasis upon diagnosis [3,18,19]. This study aimed to link compounds and molecular players evidenced in the literature that orchestrate or affect autophagy in various steps of metastasis in NB, as shown in Figure 1. The concepts presented in this figure can be categorised into four steps in cancers, including NB [21,23,25,26,49]: (1) in the pre-metastasis step, autophagy has a dual effect on promoting or suppressing this process by increasing resistance to drug therapy, including TRAIL or limiting pro-metastasis inflammatory responses, respectively; (2) during the intravasation step, autophagy can promote metastasis by inducing anoikis resistance; (3) during the extravasation step, autophagy can promote dormancy, resistance to drug therapy including TRAIL and survival of tumour cells; and (4) while in the distant metastasis site, autophagy can limit the dimension of the micrometastasis or instead promote metastatic outbreak and expansion [21,23,25,26,49]. Henceforth, the metastasis steps linked to the compounds and molecular players introduced in this review will be further analysed from the viewpoint of their specific cellular role.

ULK1, a kinase involved in autophagy, was reviewed, and it was shown that ULK1-mediated autophagy but limited apoptosis. In addition, ULK1 promoted resistance to anoikis and metastasis. Autophagy was also activated to counter TRAIL-mediated apoptosis in the NB cell lines, and collectively these three lines of evidence could be linked with steps 1–4 of the outlined steps above [32]. The role of NORAD, another oncogene in NB metastasis, was also discussed. Accordingly, NORAD may act as a suppressor of autophagic processes, and this activity suppresses genes such as Beclin 1, ATG5, and LC3B-II/I ratio and may affect nucleation, phagophore and autophagosome formation. NORAD also limited apoptosis but promoted proliferation, drug resistance and migration; therefore, it may affect steps 1–4 [33]. The effect of hsa_circ_0013401 knockdown on apoptosis and autophagy was clear since the authors demonstrated an increase in the protein levels of Bax, cleaved caspase-3, LC3B-II/I ratio, and Beclin 1, and hence this molecule may be affecting the tumour growth, proliferation and migration steps (steps 1–2 and 4) [34]. In addition, the role of SNHG16, another lncRNA, in inducing migration and autophagy through upregulating ATG5 was reviewed, and this molecule may also be affecting tumour growth, proliferation and migration steps (steps 1–2 and 4) [35]. In sum, NORAD and hsa_circ_0013401, as two oncogenes, reduced autophagy and apoptosis levels and increased metastasis, while ULK1, a mediator of autophagy, reduced apoptosis but promoted anoikis resistance and metastasis and SNHG16 induced proliferation, migration, and autophagy, revealing disparate mechanisms by which autophagy and metastasis can be linked to oncogenic function.

Furthermore, the role of MEG3 was reviewed, and it was shown that this nucleus-based lncRNA may be acting as a suppressor of autophagic processes through the axis of FOXO1, but not mTOR and this suppression affected genes such as Beclin 1 and hence may be affecting nucleation and phagophore formation, while the downregulation of MEG3 led to enhanced proliferation and migration, hence MEG3 may be limiting tumour growth, proliferation and migration steps (steps 1–2 and 4) [36]. Other tumour suppressors, including *miR-34a,* have also been investigated in the context of NB metastasis. *miR-34a* targeted ATG5 and thereby suppressed proliferation, apoptosis, metastasis and autophagy. This was evident since restoring ATG5 rescued the inhibitory effect of *miR-34a* on these processes; therefore, *miR-34a* may be affecting steps 1–2 and 4 [37]. In comparison to the oncogenes reviewed earlier, MEG3 regulated autophagy through FOXO1 modulation, but reduced metastasis through the mTOR signalling pathway [36], and *miR-34a* also suppressed autophagy and metastasis by targeting ATG5. These observations further emphasise that regulatory links between tumour suppressors, autophagy and metastasis are not clearcut and predictable [50].

Furthermore, the role of Isatin and apatinib were reviewed, and Isatin increased Beclin 1 and LC3-II levels, while apatinib increased ATG5 and LC3B-II/I ratio and apoptosis. Isatin, therefore, may be affecting the nucleation and elongation/maturation of phagophores and autophagosome formation, while apatinib may be affecting autophagosome formation and also triggering apoptosis in NB cells [38,39]. Honokiol was also shown to induce apoptosis and autophagy (leading to enhanced LC3-II levels) and reduce migration, and these effects were linked to the PI3K/Akt/mTOR pathway [40]. All three compounds reduced metastasis by inhibiting the mTOR signalling pathway and may affect tumour growth and migration steps (steps 1–2 and 4) [38,39,40].

The role of various compounds and players in inducing dormancy and the regulation of micrometastasis (steps 3–4) in terms of autophagy has been studied in other cancers [51,52] but is more limited in NB and requires further investigation.

### 3.2. The Link between the Multiple Molecular Players and Compounds with Autophagy in NB and Other Cancers, the Potential for New Biomarkers and Evidence from Clinical Trials

In terms of the significance of ULK1 as a useful biomarker for assessing NB progression and metastasis, it can be argued since ULK1 has promoted resistance to anoikis, enhanced metastasis and countered TRAIL-mediated apoptosis [32]; therefore, it may be viewed as a potential biomarker to assess NB progression and metastasis. Such evidence is largely lacking for cancers of the central nervous system but has been reported in prostate cancer. Accordingly, a study reported that elevated ULK1, a modulator of autophagy, along with mitochondrion-associated autophagy inhibitor (LRPPRC), could be used as a biochemical marker to assess cancer progression and patient OS in patients with metastatic prostate cancer [53]. This study also found that the expression of ULK1 and LRPPRC could predict multiple metastases and shorter OS [53]. Despite the existence of this data linking ULK1, autophagy and metastasis in metastatic prostate cancer, this argument in NB is still premature and is subject to further preclinical testing and clinical trials that prospectively recruit NB patients to establish such links in the face of the context-dependent autophagy roles in cancer. Furthermore, NORAD was introduced as a suppressor of autophagy that also inhibited apoptosis and assisted NB metastasis, proliferation and drug resistance [33]; hence, it can be argued that this lncRNA may be yet another suitable candidate to be utilised in NB as a biomarker of progression. As evidence, NORAD has been linked to oxaliplatin resistance in gastric cancer by enhancing autophagy levels [54]. This study found that NORAD was activated due to an increase in oxidative stress, and this lncRNA upregulated ATG5 and ATG12 levels and may be regarded as a useful biomarker in this cancer to predict oxaliplatin resistance [54]. Despite the supporting evidence offered in this prostate cancer study, the use of NORAD as a progression biomarker in NB requires a much deeper investigation at both the preclinical and clinical stages. Furthermore, hsa_circ_0013401 knockdown led to an increase in autophagy (LC3B-II/I ratio and Beclin 1); hence, this molecule suppressed autophagy. In addition, hsa_circ_0013401 enhanced tumour formation and progression through the regulatory axis of *miR-195*/PAK2 [34]; however, evidence supporting the role of this circRNA in other cancers is lacking. Finally, SNHG16 induced proliferation, migration, and autophagy through upregulating ATG5, while the sponging effect on *miR-542-3p* also contributed to this regulatory loop [35]. Consistently, the oncogenic role of SNHG16 in hepatocellular carcinoma was revealed in a study which demonstrated that this lncRNA promoted viability and autophagy but limited apoptosis to sustain resistance to sorafenib and these were collectively implemented through the axis of *miR-23b-3p*/EGR1 [55]. These links are useful but require further validation.

MEG3 as a suppressor of autophagy, reduced proliferation, migration, and invasion in NB [36]. As evidence, MEG3 overexpression has been shown to promote sensitivity to vincristine in lung cancer by inhibiting autophagy markers such as LC3-II [56]. However, this study did not suggest using this lncRNA as a biomarker for increased sensitivity to vincristine in lung cancer. In light of the two studies mentioned that both outline MEG3 as a tumour suppressor molecule, more preclinical studies are required to solidify the use of MEG3 as a useful predictor of reduced metastasis in both lung cancer and NB. 

Furthermore, *miR-34a* targeted ATG5 and thereby suppressed proliferation, apoptosis, metastasis and autophagy. This was evident since restoring ATG5 rescued the inhibitory effect of *miR-34a* on these processes [37]. In colorectal cancer, however, *miR-34a* was a target of lncRNA NEAT1. In this regulatory loop, NEAT1 targeted *miR-34a* and promoted autophagy to enable chemoresistance [57], also suggesting that *miR-34a* may be a tumour suppressor since the overexpression of *miR-34a* phenocopied NEAT1 knockdown. This role may be exploited as a potential biomarker in the future.

Isatin, a compound that has linked autophagy to NB metastasis, effectively reduced NB metastasis by activating autophagy and modulating signalling pathways such as mTOR [38]. Evidence supporting the linkage between Isatin, autophagy and metastasis in other cancers is largely lacking. Despite this, studies have suggested that Isatin may suppress metastasis in NB through the regulation of reactive oxygen species, vascular endothelial growth factor receptor 1, chemokines, and other signalling molecules [58]. Therefore, the effective suppression of NB metastasis by modulating autophagy using Isatin is significant and warrants further investigation and study in both NB and other cancers. 

For apatinib, as a compound that has linked NB metastasis to autophagy, it can be stated that this compound induced autophagy while reducing metastasis levels [39]. This evidence is supported by a study in human papillary thyroid carcinoma, in which apatinib induced autophagy and apoptosis and limited tumour growth through the PI3K/AKT/mTOR pathways [59]. Given this background, apatinib’s effective suppression of NB and papillary thyroid carcinoma through autophagy is significant and warrants further investigation and study in both cancers. 

Finally, Honokiol was also shown to induce apoptosis and autophagy (increase in LC3-II) and reduce migration, and these effects were linked to the PI3K/Akt/mTOR pathway [40]. This exact effect has been obtained in a study in osteosarcoma in which this compound induced autophagy and apoptosis through the PI3K/Akt/mTOR pathway [60], suggesting that this molecule may be a useful drug for inhibiting metastasis linked to autophagy in both cancer types.

**Figure 8:**
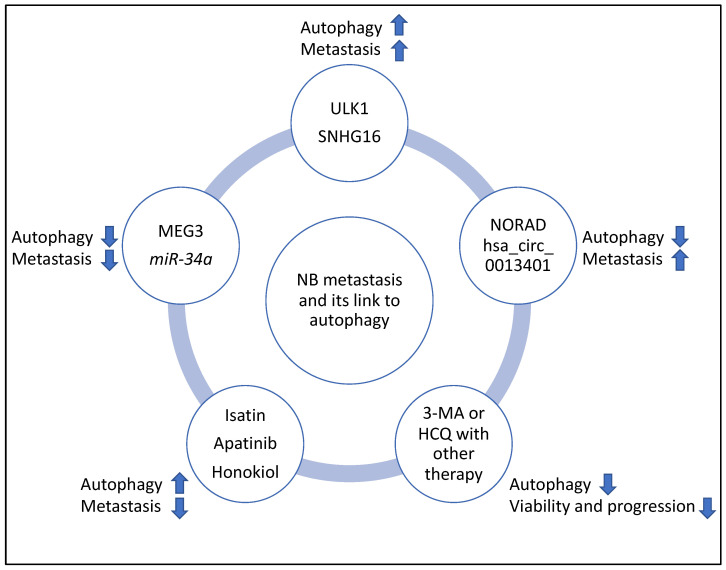
Summary flowchart of the molecular players and compounds reviewed in this study linking NB metastasis to autophagy. ULK1 and SHNG16 induced autophagy, metastasis, and MEG3 and *miR-34a* suppressed autophagy and metastasis; NORAD and hsa_circ_0013401 suppressed autophagy but induced metastasis, and Isatin, Apatinib and Honokiol induced autophagy but suppressed metastasis. Finally, 3-MA and HCQ may be useful inhibitors of autophagy to improve the efficacy of therapy in NB (for example, the combination of 3-MA with sulforaphane or the combination of HCQ with vincristine, reduced viability and progression of NB, respectively) and clinical trials may be launched to further investigate these links. It is noteworthy that in this figure, the arrows pointing upwards and downwards signify induction or suppression, respectively.

In addition, the clinicaltrials.gov website (https://clinicaltrials.gov, accessed on 10 March 2023) was searched for studies that may link the regulation of autophagy with NB progression; however, currently, to the best of the author’s knowledge, such studies are largely lacking. Despite this, other studies have suggested that autophagy levels increased following chemotherapy. Therefore, using autophagy inhibitors such as 3-Methyladenine (3-MA) and Hydroxychloroquine (HCQ) could limit tumour proliferation and progression and may justify designing clinical trials aiming to use these compounds to improve the efficacy of NB chemotherapy [41,42]. Specifically, the combination of 3-MA with sulforaphane or the combination of HCQ with vincristine reduced the viability and progression of NB cells, respectively [41,42]. It is noteworthy that 3-MA limits autophagosome formation, while HCQ reduces autophagosome to lysosome fusion and autophagic flux, and the latter molecule is the only compound that has been clinically approved for autophagy inhibition [61,62]. Finally, the summary flowchart of the molecular players and compounds reviewed in this study linking NB metastasis to autophagy is shown in Figure 8.

In conclusion, this study reviewed and analysed multiple compounds or molecular players affecting autophagy and their link to the multiple stages of NB metastasis. It was found that the induction or suppression of autophagy by these multiple molecular players and compounds was context-dependent; they linked autophagy to metastasis in NB and may be utilised as potential biomarkers to predict metastasis or for NB treatment given that sufficient preclinical and clinical investigations are carried out. Studying these molecules and their associated processes may lead to developing new treatment options and fine-tuning current therapeutics that will ultimately positively impact the quality of life of NB patients.

## Figures and Tables

**Table 1:** The list of the 11 molecules and compounds linking autophagy to metastasis in NB.

Compounds or Molecule	Cancer Type	Effect on Autophagy	Effect on Metastasis	Reference
ULK1	NB	Mediated autophagy since SBI-0206965 (an inhibitor of ULK1) reduced autophagic flux and LC3 lipidation	Promoted metastasis	[32]
NORAD	NB	Suppressed autophagy since in NORAD knockdown, Beclin 1, ATG5, and LC3B-II/I ratios were increased	Promoted metastasis	[33]
hsa_circ_0013401	NB	Suppressed autophagy since hsa_circ_0013401 knockdown led to increased LC3B-II/I ratio and Beclin 1 levels	Promoted metastasis	[34]
SNHG16	NB	Induced autophagy through upregulating ATG5	Promoted metastasis	[35]
MEG3	NB	Suppressed autophagy, since silencing of MEG3 increased autophagy markers including ATG16, Beclin 1, and ATG3	Suppressed metastasis	[36]
*miR-34a*	NB	Suppressed autophagy since *miR-34a* negatively targeted ATG5	Suppressed metastasis	[37]
Isatin	NB	Induced autophagy since Isatin increased Beclin 1, and LC3-II levels	Suppressed metastasis	[38]
Apatinib	NB	Induced autophagy since Apatinib increased ATG5 and LC3B-II/I ratio	Suppressed metastasis	[39]
Honokiol	NB	Induced autophagy since Honokiol increased LC3-II levels	Suppressed metastasis	[40]
3-Methyladenine (3-MA) with sulforaphane	NB	Suppressed autophagy	Reduced tumour viability	[41]
Hydroxychloroquine (HCQ) with vincristine	NB	Suppressed autophagy	Reduced tumour progression	[42]

## Data Availability

Data sharing is not applicable.

## Abbreviations

ALT	Alternating length of telomeres
AMPK	AMP-activated kinase
ATGs	Autophagy-related genes
DOX	Doxorubicin
EFS	Event-free survival
EMT	Mesenchymal transition
HCQ	Hydroxychloroquine (HCQ)
HDAC8	Histone deacetylase 8
IC50	Half-maximal inhibitory concentration
IDRF	Image-defined risk factors
INRGSS	International neuroblastoma risk group system
INSS	International neuroblastoma staging system
LC3	microtubule-associated protein light chain 3
LncRNAs	Long non-coding RNAs
LRPPRC	Mitochondrion-associated autophagy inhibitor
3-MA	3-Methyladenine
NB	Neuroblastoma
NSG	NOD/SCID-gamma
OS	Overall survival
PARP	Poly-ADP ribose polymerase
TERT	Telomere reverse transcriptase
TRAIL	TNF-related apoptosis-inducing ligand
VEGFR-2	Vascular endothelial growth factor receptor 2
ULK1	UNC-51-like kinase1
